# Purification, Crystallization and Preliminary X-ray Crystallographic Studies of RAIDD Death-Domain (DD)

**DOI:** 10.3390/ijms10062501

**Published:** 2009-06-03

**Authors:** Tae-ho Jang, Hyun Ho Park

**Affiliations:** Department of Biochemistry, School of Biotechnology at Yeungnam University, Gyeong-san, Korea

**Keywords:** RAIDD, PIDDosome, crystallization, Death-Domain (DD)

## Abstract

Caspase-2 activation by formation of PIDDosome is critical for genotoxic stress induced apoptosis. PIDDosome is composed of three proteins, RAIDD, PIDD, and Caspase-2. RAIDD is an adaptor protein containing an *N*-terminal Caspase-Recruiting-Domain (CARD) and a *C*-terminal Death-Domain (DD). Its interactions with Caspase-2 and PIDD through CARD and DD respectively and formation of PIDDosome are important for the activation of Caspase-2. RAIDD DD cloned into pET26b vector was expressed in *E. coli* cells and purified by nickel affinity chromatography and gel filtration. Although it has been known that the most DDs are not soluble in physiological condition, RAIDD DD was soluble and interacts tightly with PIDD DD in physiological condition. The purified RAIDD DD alone has been crystallized. Crystals are trigonal and belong to space group P3_1_21 (or its enantiomorph P3_2_21) with unit-cell parameters *a* = 56.3, *b* = 56.3, *c* = 64.9 Å and *γ* = 120°. The crystals were obtained at room temperature and diffracted to 2.0 Å resolution.

## 1. Introduction

Death-Domains (DDs) are protein interaction modules composed of six-helix bundle. DDs, together with DEDs, CARDs, and PYDs, comprise the DD superfamily and has a pivotal role in apoptosis signaling pathway by mediating homotypic interaction [1,2]. Apoptosis signaling pathway is mediated by sequential activation of caspases that is cysteinyl protease. Activation of caspases is mediated by large molecular complexes in the apoptosis signaling pathway [3,4,5,6].

PIDDosome, caspase-2 activating large molecular complex, is composed of three different protein components including PIDD (p53-induced protein with a DD), RAIDD (RIP-associated ICH-1 homologous protein with a death domain), and caspase-2 [5]. Caspase-2 activation by PIDDosome is critical for genotoxic stress induced apoptosis [7]. PIDD contains 910 residues with seven leucine rich repeats (LRRs), two ZU-5 domains and a *C*-terminal Death Domain (DD) [8]. PIDD is essential for cell death, which it facilitates by activating caspase-2. In addition, PIDD is also critical for cell survival due to its interaction with RIP1, a kinase that has been implicated in the activation of NF-κB [7]. The results of several studies indicate that PIDD may be a molecular switch that controls the balance between life and death upon genotoxic stress [7]. Caspase-2 is the second caspase to be identified and is the most evolutionarily conserved caspase cross the species of animal [9]. RAIDD, an adaptor protein containing both an N-terminal CARD and a C-terminal DD, interacts to Caspase-2 and PIDD through CARD-CARD and DD-DD respectively [10,11]. Despite the fundamental importance of the Death-Domain superfamily in apoptotic and inflammatory signaling pathways, limited crystal structures are available [12].

As the first step toward elucidating molecular structure of PIDDosome and further understanding homotypic interaction of DD in apoptosis signaling pathway, we over-expressed, purified and crystallized RAIDD DD. Although it has been known that most DDs are not soluble under physiological conditions [13,14], RAIDD DD was soluble and interacts tightly with PIDD DD under physiological conditions. Crystals are trigonal and belong to space group P3121 with unit-cell parameters a = 56.3, b = 56.3, c = 64.9 Å and γ= 120°. The crystals were obtained at room temperature and diffracted to 2.0 Å resolution. Details of the structure of RAIDD DD should enable us to understand the mechanism of the PIDDosome formation via DD:DD interaction.

## 2. Results and Discussion

### 2.1. Over-expression and purification of RAIDD DD

**Figure 1.**
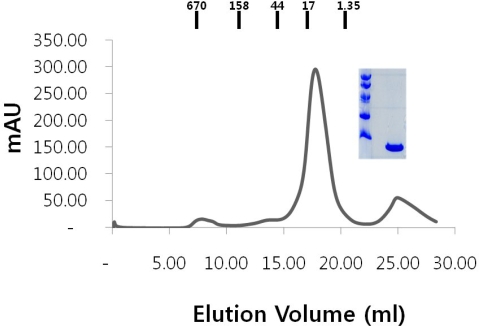
Purification of the RAIDD DD. The profile showing the elution of the RAIDD DD on Gel-filtration chromatography. SDS-PAGE (15% gel) of the purified RAIDD DD.

As described below in the Experimental section, a DNA fragment encoding the RAIDD DD (94–199) was cloned by the polymerase chain reaction (PCR) using a plasmid DNA containing the full-length RAIDD gene as the template in order to elucidate the molecular structure of RAIDD DD by the X-ray protein crystallography. Ligation of the PCR product to the expression vector pET-26b produces plasmid pET26RAIDD-DD. This vector construction adds an eight-residue tag including C-terminal hexahistidine which has been used as efficient tag for affinity chromatography. The resulting plasmid was transformed into BL21 (DE3) *E. coli* competent cells and expressed by treating the bacteria with 0.5 mM isopropyl-D-thiogalactopyranoside (IPTG). After Ni-NTA affinity chromatography followed by gel-filtration chromatography, we could obtain pure RAIDD DD protein (Figure 1). The main peak that contains RAIDD DD eluted around 10 kDa position. It indicates RAIDD DD is existed as a monomer in solution.

RAIDD DD was concentrated to 4–6 mg mL^−1^ using an Millipore concentration kit (Millipore) for crystallization trials. The selenomethionine-substituted RAIDD DD was expressed in the methionine auxotrophic strain B834 (Novagen) grown in minimal medium supplemented with seleno-l-methionine (Sigma) and other nutrients. It was purified and crystallized using the same procedure as used for the native protein.

### 2.2. Crystallization of RAIDD DD

**Figure 2.**
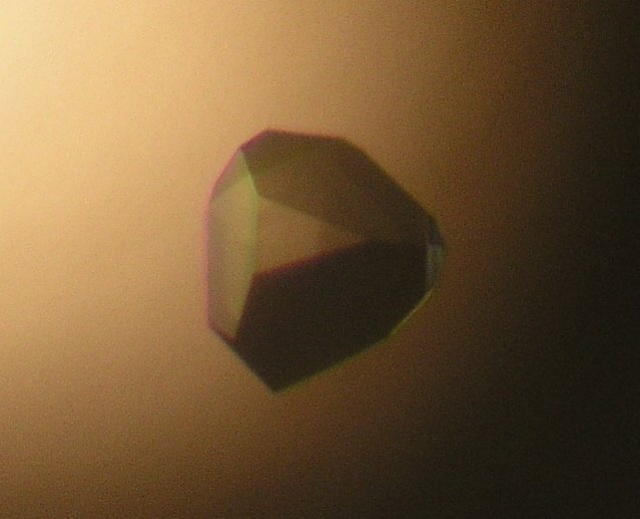
Crystal of RAIDD DD. A native RAIDD DD crystal grown in three days in the condition of 2 M Na/K phosphate at pH 7.0. Its approximate dimentions are 0.2 X 0.2 X 0.2 mm.

Crystallization conditions were initially screened at 297K by the hanging-drop vapor-diffusion method using screening kits from Hampton Research (Crystal screening I and II, Natrix, MembFac, SaltRX) and from deCODE Biostructures Group (Wizard I and II). Crystals were grown on a siliconized cover slip by equilibrating a mixture containing 1 μL of protein solution (4–6 mg·mL^−1^ protein in 20 mM Tris-HCl at pH 8.0, 150 mM NaCl) and 1 μL of a reservoir solution (2 M Na/K phosphate at pH 7.0) against 0.5 ml of reservoir solution. Crystals appeared in three days and grew to a maximum dimension of 0.2 × 0.2 × 0.2 mm (Figure 2). The selenomethionine-substituted RAIDD DD crystals were also grown under the similar condition (2.1 M Na/K phosphate at pH 7.0).

### 2.3. Preliminary X-ray diffraction studies

For diffraction experiments, crystals were transiently soaked in a solution corresponding to the reservoir solution but supplemented with 20% (v/v) glycerol. The crystals were cryocooled at 110K. A single-wavelength anomalous diffraction (SAD) data set was collected at the selenium peak wavelength (E=12664ev, λ=0.979Å) at the X4A beamline of National Synchrotron Light Source (NSLS). Selenium labeled methionine will be found and the structure will be phased using the program SOLVE/RESOLVE [15]. Data processing and scaling was carried out in the HKL2000 package [16]. A native data set was collected and used for model refinement using CNS [17]. The data-collection statistics are summarized in Table 1. The structure was determined and deposited at protein data bank (PDBID: 2O71) [19].

### 2.4. Functional test of RAIDD DD

**Figure 3.**
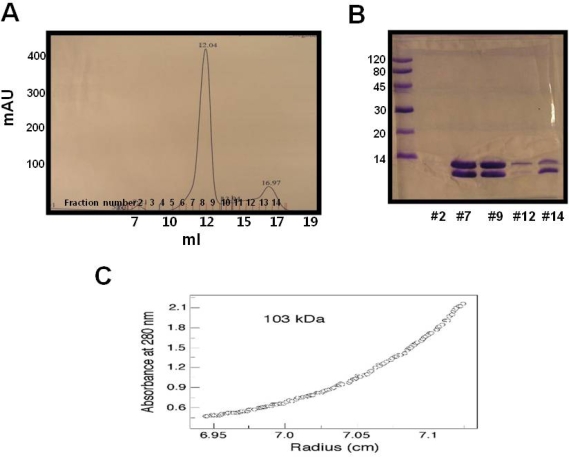
Functional test of RAIDD DD. A. Profile of Gel-filtration chromatography. Elution volume around 12 mL indicates molecular weight of 120 kDa; B. 15 % SDS-PAGE showed that the peak from profile of gel-filtration chromatography is a complex. Fraction #12 and #14 showed that uncomplexed left over proteins; C. The equilibrium radial absorbance profiles at 25,000 rev./min by analytical ultracentrifugation analysis for RAIDD DD: PIDD DD complex.

DDs mediate protein-protein interaction. RAIDD DD is protein interaction module that is involved in the interaction to PIDD DD to form a PIDDosome. This homotypic interaction is critical for caspase-2 activation and genotoxic stress induced apoptosis. To test the function of purified RAIDD DD, we purified PIDD DD and analyzed binding ability of RAIDD DD to PIDD DD. Gel-filtration profile and SDS-PAGE clearly showed that RAIDD forms a tight complex with PIDD DD (Figure 3A, 3B). The absolute molecular mass of 103 kDa (Figure 3C), determined by analytical equilibrium ultracentrifugation, indicates that the stoichiometry of the RAIDD DD: PIDD DD complex is 4:4 or 5:5.

## 3. Experimental Section

### 3.1. Expression and purification

The construct for expression of human RAIDD DD (94–199) was made as follows. The cDNA of full length RAIDD DD was used as a template for the polymerase chain reaction (PCR) and the plasmid vector pET26b (Novagen, USA) was used to add a hexahistidine tag at the carboxy-terminus of RAIDD DD for affinity purification. PCR products were digested with *NdeI* and *XhoI* (New England Biolabs, USA) restriction enzymes and ligated into pET26b. This vector construction adds an eight-residue tag including C-terminal hexahistidine (LEHHHHHH).

The resulting plasmid was transformed into BL21 (DE3) *E. coli* competent cells. The expression was induced by treating the bacteria with 0.5 mM isopropyl β-d-thiogalactopyranoside (IPTG) for overnight at 293 K. The bacteria were then collected, resuspended and lysed by sonication in 50 mL lysis buffer (20 mM Tris-HCl at pH 7.9, 500 mM NaCl, 5 mM imidazole). The bacterial lysate was then centrifuged at 14,000 g for 1 hr at 277 K. The supernatant fraction was applied to gravity-flow column (Bio-Rad, USA) packed with Ni-NTA affinity resin (Qiagen, USA). The unbound bacterial proteins were removed from the column using washing buffer (20 mM Tris-HCl at pH 7.9, 500 mM NaCl, 60 mM imidazole and 10% glycerol). The C-terminal His6-tagged RAIDD DD was eluted from the column using an elution buffer (20 mM Tris-HCl at pH 8.0, 500 mM NaCl, 250 mM imidazole). The protein purity was further improved by using a Superdex 200 gel filtration column 10/30 (Pharmacia) which was pre-equilibrated with a solution of 20 mM Tris-HCl at pH 8.0 and 150 mM KCl.

RAIDD DD was concentrated to 4–6 mg mL^−1^ using a Millipore concentration kit (Millipore, USA) for crystallization trials. The selenomethionine-substituted RAIDD DD was expressed in the methionine auxotrophic strain B834 (Novagen, USA) grown in minimal medium supplemented with seleno-l-methionine (Sigma, USA) and other nutrients. It was purified and crystallized using the same procedure as used for the native protein.

### 3.2. Crystallization

Crystallization conditions were initially screened at 297 K by the hanging-drop vapor-diffusion method using screening kits from Hampton Research (Crystal screening I and II, Natrix, MembFac, SaltRX) and from deCODE Biostructures Group (Wizard I and II). Crystals were grown on a siliconized cover slip by equilibrating a mixture containing 1 μL of protein solution (4–6 mg mL^−1^ protein in 20 mM Tris-HCl at pH 8.0, 150 mM NaCl) and 1 μL of a reservoir solution (2M Na/K phosphate at pH 7.0) against 0.5 mL of reservoir solution. Crystals appeared in three days and grew to a maximum dimension of 0.2 × 0.2 × 0.2 mm (Figure 1). The selenomethionine-substituted RAIDD DD crystals were also grown under the similar condition (2.1 M Na/K phosphate at pH 7.0).

### 3.3. Data collection and analysis

For diffraction experiments, crystals were transiently soaked in a solution corresponding to the reservoir solution but supplemented with 20% (v/v) glycerol. The crystals were cryocooled at 110 K using a nitrogen stream (Cryocool, Cryo Industries, New Hampshire, USA). A single-wavelength anomalous diffraction (SAD) data set was collected at the selenium peak wavelength (E=12664ev, λ=0.979Å) at the X4A beamline of National Synchrotron Light Source (NSLS). Data processing and scaling was carried out in the HKL2000 package [16]. A native data set was collected and used for model refinement. The data-collection statistics are summarized in Table 1.

### 3.4. Gel filtration chromatography

Purification method for PIDD DD is described at Park *et al*. [18]. Separately purified and quantified RAIDD DD, PIDD DD were incubated for 1hr at room temperature. Following pre-incubation, the mixture was concentrated to 15 ~ 20 mg mL^−1^ using a concentration kit (Millipore, USA). The concentrated protein mixture was then applied to a Superdex 200 gel-filtration column 10/30 (GE healthcare, USA), which was pre-equilibrated with a solution of 20 mM Tris-HCl at pH 8.0 and 50 mM NaCl. Formation of the complex was then detected by evaluating the positions of the eluted peak followed by SDS-PAGE.

### 3.5. Ultracentrifugation

Analytical ultracentrifugation experiments were performed using a Beckman XL-1 analytical ultracentrifuge at 298 K. Absorbance was measured at the maximum wavelength as a function of radius at 25,000 rev./min.

## 4. Conclusions

In the current study, the RAIDD DD (94–199) of human RAIDD was over-expressed and purified. The recombinant RAIDD DD was fully functional by tight binding to PIDD DD which is well known binding partner. The purified RAIDD DD alone has been crystallized. Crystals are trigonal and belong to space group P3_1_21 with unit-cell parameters a = 56.32, b = 56.32, c = 64.95 Å and γ = 120°. The crystals were obtained at room temperature and diffracted to 2.0 Å resolution.

## Figures and Tables

**Table 1.** Crystallographic statistics.

**Data collection**	**Se-Met**	**Native**
Space group	P3_1_21	P3_1_21
Cell dimensions		
*a*, *b*, *c*	56.3Å, 56.3Å, 64.9Å	56.1Å, 56.1Å, 64.9Å
Resolution	50–2.0Å	50–2.0Å
†*R*_sym_	6.2% (27.4%)	5.5% (17.9%)
†*I*/σ(*I)*	17.2 (2.8)	14.5 (2.3)
†Completeness	100.0% (100.0%)	99.7% (99.8%)
†Redundancy	11.0 (10.6)	8.9 (8.9)

**Refinement**	
Resolution	50–2.0Å
No. reflections used (completeness)	8063 (96.9%)
*R*_work_/*R*_free_	23.1%/24.1%
No. atoms	
Protein	704
Water and other small molecule	55
Average B-factors	
Protein	32.0 Å^2^
Water and other small molecule	40.4 Å^2^
R.m.s deviations	
Bond lengths	0.005Å
Bond angles	1.0°
Ramachandran Plot	
Most favored regions	91.3%
Additional allowed regions	8.7%

† Highest resolution shell is shown in parenthesis.
